# Chromosomal catastrophe is a frequent event in clinically insignificant prostate cancer

**DOI:** 10.18632/oncotarget.4900

**Published:** 2015-08-21

**Authors:** Irina V. Kovtun, Stephen J. Murphy, Sarah H. Johnson, John C. Cheville, George Vasmatzis

**Affiliations:** ^1^ Department of Molecular Pharmacology and Experimental Therapeutics, Mayo Clinic, Rochester, Minnesota, USA; ^2^ Department of Molecular Medicine, Mayo Clinic, Rochester, Minnesota, USA; ^3^ Department of Laboratory Medicine and Pathology, Mayo Clinic, Rochester, Minnesota, USA; ^4^ Department of Center of Individualized Medicine, Mayo Clinic, Rochester, Minnesota, USA

**Keywords:** chromothripsis, catastrophe, genomic rearrangements, prostate cancer, gleason score

## Abstract

Massive genomic rearrangements, a result of single catastrophic event termed chromothrispsis or chromosomal catastrophe, have been identified in a variety of human cancers. In a few cancer types, chromothripsis was found to be associated with poor prognosis. We performed mate-pair sequencing and analysis of structural rearrangements in 132 prostate cancer cases which included clinically insignificant Gleason score 6 tumors, clinically significant tumors of higher grade (7+) and high grade prostatic intraepithelial neoplasia. Chromothripsis was observed at least 30 per cent of the samples across different grades. Surprisingly, it was frequently observed in clinically insignificant Gleason score 6 tumors, indicating that chromothripsis does not define more aggressive phenotype. The degree of chromothripsis did not increase significantly in tumors of advanced grades and did not appear to contribute to tumor progression. Our data showed that distribution of chromothriptic rearrangements differed from that of fragile sites but correlated with the size of chromosomes. We also provided evidence that rearrangements resulting from chromothripsis were present in the cells of neighboring Gleason patterns of the same tumor. Our data suggest that that chromothripsis plays role in prostate cancer initiation.

## INTRODUCTION

Consequences of chromothripsis or chromosomal catastrophe, a recently described phenomenon, have been observed in many tumor types [1]. Complex clustered DNA rearrangements identified by means of massive parallel sequencing are considered to be a hallmark of chromothripsis, and are believed to significantly contribute to tumorigenesis. The degree of chromothripsis has been reported to vary among different cancers [1–5]. Chromothripsis incidence of 10% was shown in chronic lymphocytic leukemia [1–2], while a frequency as high as 33% was observed in osteosarcoma [1–2]. A few studies have shown a correlation between the prevalence of clustered rearrangements in patients’ tumor and their survival suggesting that chromothripsis defines more aggressive cancer. For example, chromothripsis has been strongly linked to poor survival in acute myeloid leukaemia, neuroblastoma and multiple myeloma [4–7]. Careful analysis of the cells from tumors that show chromothripsis using FISH, revealed that nearly the entire cell population harbor clustered rearrangements, indicating that chromothripsis is a relatively early event [8]. Analysis of breakpoint junctions within clusters provided clues on the type of rearrangements and on possible mechanisms underlying DNA repair following chromothripsis. Notably, the copy number states within chromothriptic regions were often low and fluctuated between one or two, rarely three [8,9]. Examination of the breakpoints at the sites of chromothripsis showed that duplications, deletions and insertions were present [1–2]. This observation led to the conclusion that the mechanism for rejoining shattered pieces of DNA is likely replication-dependent [8,9].

Complex rearrangements resulting from minicatastrophe's have also been described in germline DNA [10–12]. The majority of these events involved multiple chromosomes, showed little homology at the breakpoints and represented alterations with copy neutral changes, often including balanced translocations and inversions [10, 12, 13]. Although, limited numbers of cases have been evaluated so far, the incidence of chromothriptic events in germline DNA from subjects with constitutional diseases was high, ranging from 19.2% [10] to 80% [12].

Structural DNA alterations are believed to play a role in initiation and progression of prostate cancer (PCa). Several studies have profiled PCa DNA using deep sequencing to characterize entire landscapes of genomic rearrangements [14,15]. By far the most recurrent event reportedly involves a fusion between androgen-regulated promoter of *TMPRSS2* gene and *ERG* oncogene due to somatic deletion on chromosome 2, effecting 50–60% of PCa cases [16,17]. Another frequently observed alteration is disruption of tumor suppressor *PTEN* [14,18]. Complex clustered rearrangements are frequently observed in PCa [15,19]. The term chromoplexy was introduced to describe the phenomenon of genome restructuring and was suggested to be a result of accumulation of numerous discrete events during prostate carcinogenesis [15].

Chromothripsis was also reported in a few cases of PCa [20,21] The incidence of chromothripsis in PCa and its possible contribution to tumor progression, however, have not been examined. In this study we have analyzed landscape of structural rearrangements in a large set of PCa from radical prostatectomy specimens that included clinically insignificant Gleason score 6 (GS6) and clinically significant (GS7 and higher) tumors. In order to gain an insight into contribution of chromothripsis to PCa initiation and progression we estimated frequency of chromothripsis, its association with Gleason grade, *ERG* status and distribution of fragile sites.

## RESULTS

### Incidence of chromothripsis in prostate cancer cases

**Figure 1:**
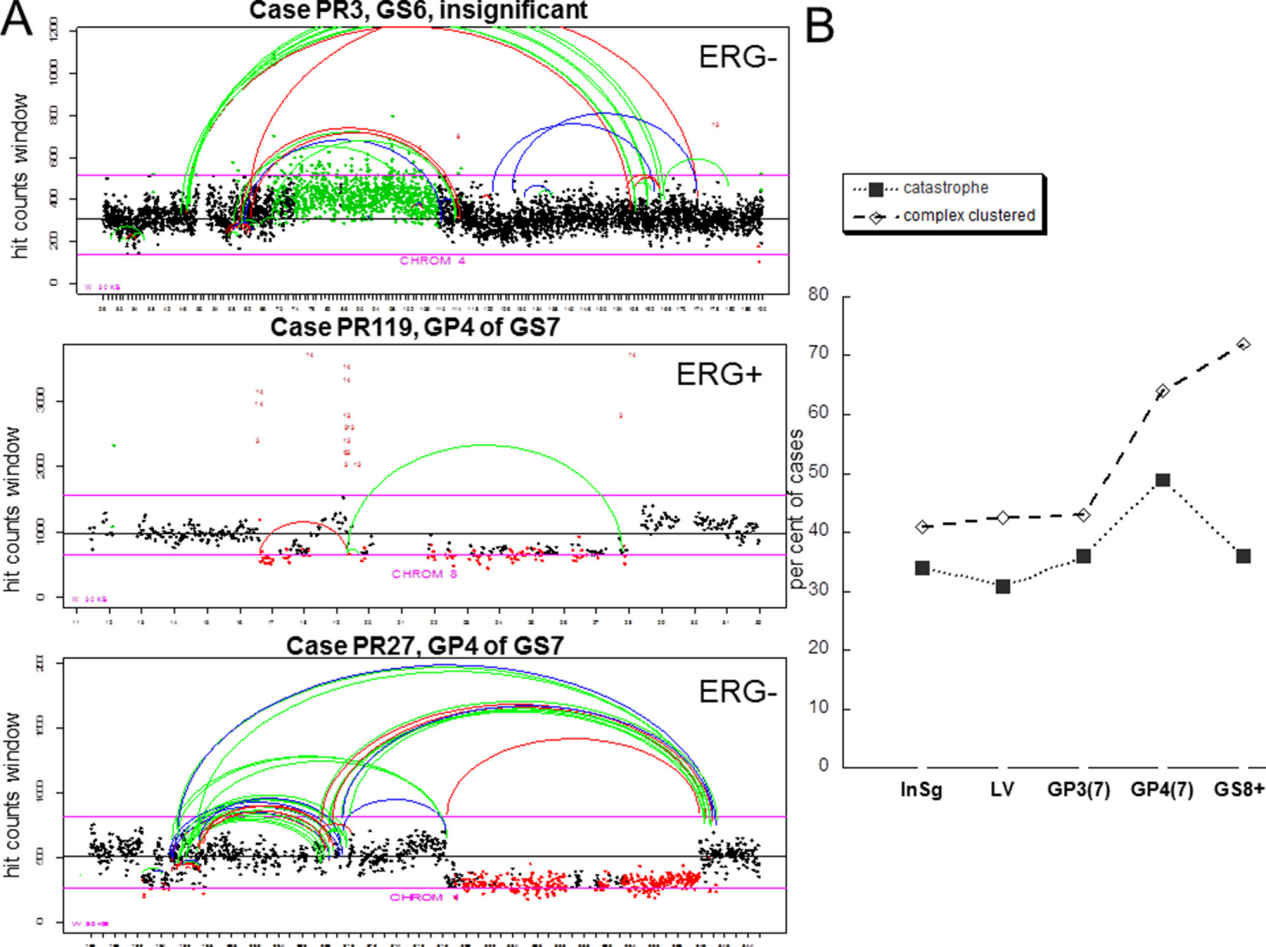
Incidence of chromothripsis and complex inter-chromosomal clustered breaks **A.** Count plots showing frequency distribution of reads in 30 Kb windows and localization of breakpoints for indicated chromosome. The X axis spans the length of the chromosome, the Y axis shows the number of reads for each window. Window counts are shown by points colored according to the prediction of CNV algorithm. Black points are normal, red points correspond to deletions and green points show gains. Color of the connecting loops indicate polarity of the joined chromosomal pieces: red shows forward direction (concordant) for both pieces (represents deletions), green indicates switch in polarity (represents inversion) and blue indicates change in direction (gain). Inter-chromosomal events are shown in red, numbers correspond to other involved chromosomes. X is coded as chromosome 23 and Y is coded as chromosome 24. Representatives of chromosomal catastrophe (top and bottom panels) and complex inter-chromosomal clustered rearrangements (middle panel) are shown. **B.** Incidence of catastrophic events and complex inter-chromosomal rearrangements in prostate cancer pathologic groups. InSg is clinically insignificant GS6, LV is large volume GS6, GP3 and GP4 are Gleason pattern 3 and 4 of GS7 respectively.

**Figure 2:**
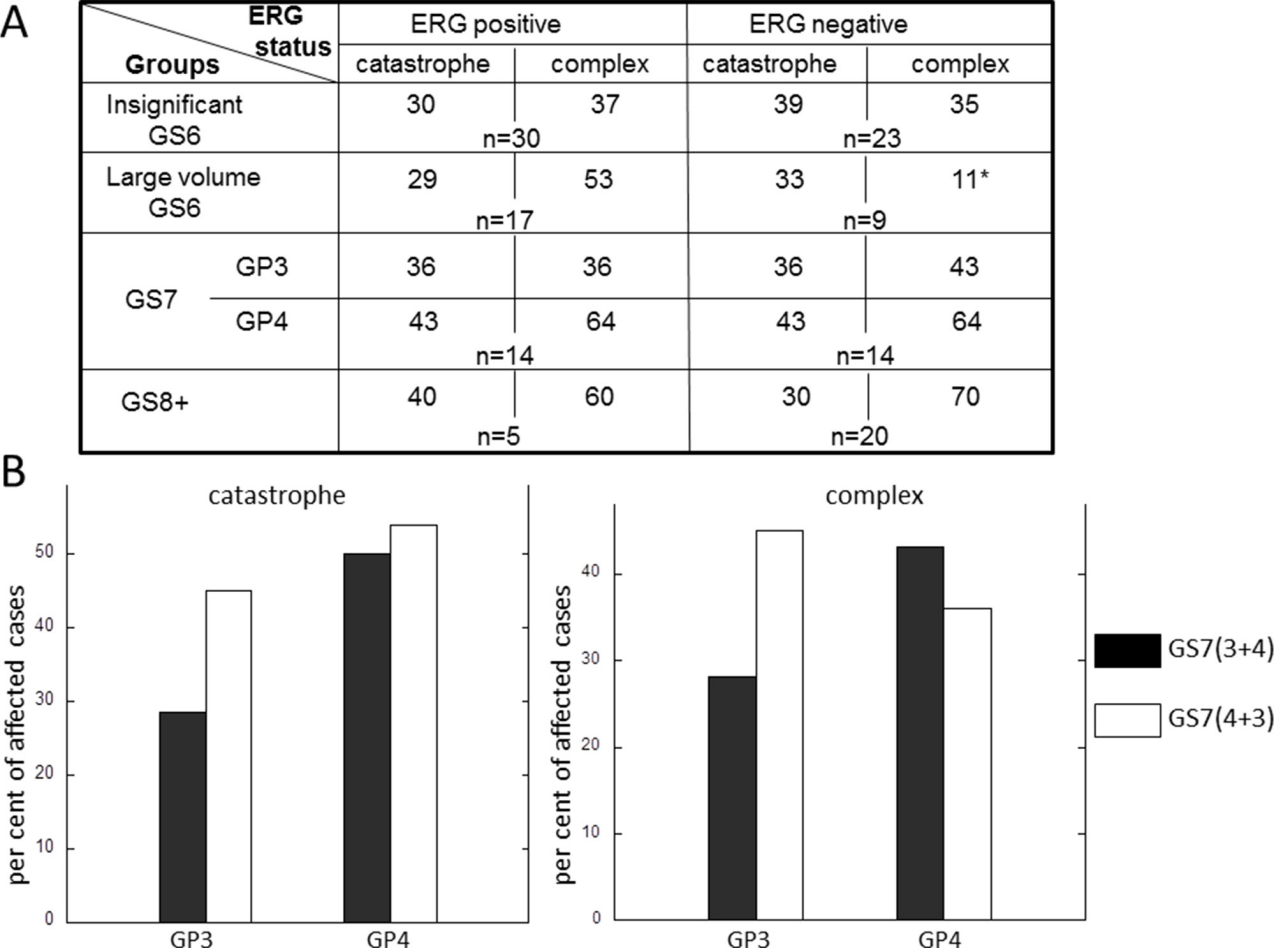
Frequency of chromothripsis and complex inter-chromosomal clustered breaks in adjacent tumors of different *ERG* status **A.** Chromothriptic events and inter-chromosomal clustered breaks for *ERG* positive and *ERG* negative prostate tumors. **B.** Comparison of incidence of chromothripsis and complex inter-chromosomal clustered breaks in GP3 and GP4 tumors originating from GS7 (3+4) GS7 (4+3).

Using mate pair next generation sequencing protocol in conjunction with bioinformatics analysis [22,23] we characterized a landscape of structural rearrangements in total of 132 PCa. The cases were grouped according to pathology description into insignificant GS6 (confined, tumor volume <0.6 cm3) (*n* = 53), large volume GS6 (>1.0 cm3) (*n* = 26), GS7 (*n* = 28) and GS8+ (*n* = 25) (consisting of GS8 and GS9, 4 and 21 cases respectively) tumors. Adjacent Gleason pattern, GP3 and GP4, tumors from each GS7 case were collected and analyzed separately [19, 23]. Cells from HGPIN associated with the tumor were also collected for a subset of these cases (total of 38) and analyzed. Sixteen of those were associated with insignificant GS6, six – with large volume GS6, five- with GS7 and eleven-with GS8+. Clustered breakpoints were classified as chromothripsis events if the following criteria were met: 1) affected locus comprised a region exceeding 10 Mb, 2) a number of clustered breakpoints within the region was higher than dozen, 3) breakpoints comprising the cluster involved one or two chromosomes and 4) included alternating copy number states, insertions and loss of heterozygositiy (Figure 1A, top and bottom panels), consistent with features described previously [1,8,9]. The events with breakpoints comprising a cluster that involved more than two chromosomes and harbored at least 7 alterations were classified as complex inter-chromosomal clusters (Figure 1A, middle panel, genome plot in Supplementary Figure S1), consistent with the phenomenon termed chromoplexy [15]. Both chromosomal catastrophe and complex inter-chromosomal clusters were observed in all analyzed groups (Figure 1B). The incidence of chromothripsis in every group was relatively high, with 30–45% of cases meeting criteria for at least one catastrophic event (Figure 1B). Surprisingly, chromosomal catastrophe was present in clinically insignificant disease (Figure 1B), affecting 34% of cases. Similar fraction of cases was affected in large volume GS6 and in GP3 tumors adjacent to GP4, 31% and 36% respectively, of GS7. Thus, no association was observed between incidence of chromothripsis and significance/aggressiveness of the tumor when GP3s from insignificant GS6, large volume GS6 and GS7 were compared (Figure 1). We next split GS7 group into GP3+4 and GP4+3 and analyzed incidence of chromothripsis in GP3 and GP4 tumors of these subgroups (Figure 2A). The per cent of affected cases in GP3 from GS7(4+3) was closer to its counterpart GP4 than to GP3 from GS7 (3+4). However, the difference between these two subsets is likely to be insignificant (28% in GS7 (3+4) versus 45% in GS7(4+3)) since the number of cases in each subgroup was relatively small (14 and 11 respectively). Consistent with this is the incidence of chromothripsis in GS8+ group, at 36% level, where most of the cases (21 out of 25) were GS9. Collectively, these data suggest that chromothripsis is not critical for cancer progression.

The landscape of genomic rearrangements was also assessed in HGPINs associated with some of the analyzed tumors. Most of the HGPINs harbored very few rearrangements (representative example is shown in Supplementary Figure S2), and none showed chromothripsis, unlike their corresponding tumors (Supplementary Figure S2). The results suggested that HGPIN adjacent to the cancer had minimal if any shared genomic relationship to the invasive tumor, therefore, unlikely was the precursor lesion in these tumors. Other significant genomic alterations must be required for an invasive phenotype beyond that seen in HGPIN.

### Chromothripsis and common drivers in prostate cancer

We next compared clinical outcome (systemic progression and/or death from PCa) of cases that showed presence of chromothripsis to that of cases that did not. No differences in outcome was found between the two groups. As expected no events were observed in insignificant GS6 group (Supplementary Table S1). Only four cases in GS7 group (three in 3+4 and one in 4+3 subset) had systemic progression (no PCa related death) with one case being affected by chromothripsis. Roughly, half of the cases in GS8+ group that developed systemic progression were affected by chromothripsis (four out of 7) or died of the disease (one out of three). Although, the number of outcome events across the groups was small, no evidence of chromothripsis being associated with most aggressive PCa was found.

Alterations at *ERG*, *PTEN* and *c-myc* genes are frequently observed in PCa, and considered to drive disease progression [14,16,24] None of these loci was affected by chromothripsis in insignificant GS6, large volume GS6 or GP3 associated with GP4 in GS7 cancer. In one GS7 case catastrophe involving *c-myc* gene was observed in GP4 but not in GP3. The corresponding alteration, however, did not result in amplification of *c-myc* which is believed to contribute to oncogenic properties and to drive progression. Similarly, no case showed chromothripsis at chromosome 21 involving *ERG* gene, known to be rearranged on average in 60% of PCa cases [16,17]. In a substantial number of cases catastrophe was present on several different chromosomes (Supplementary Table S2). Approximately ten per cent of cases in insignificant GS6 showed catastrophe on 2 or 3 chromosomes (4 and 1 case respectively). This observation is consistent with the notion that the affected loci are unlikely to involve genes driving tumor progression. Our data rather suggest that alterations comprising chromothriptic cluster affect genes that play role in cancer initiation.

Common genetic alteration in prostate cancer, *TMPRSS2-ERG* fusion, is believed to be an early event in prostate carcinogenesis. It was previously reported to be present in 19% of HGPINs and suggested to occur at transition between benign and PIN epithelium [25]. Since chromothripsis is observed early in prostate tumorigenesis, and is present in 30% of insignificant GS6 cancers (Figure 1B), we next examined, whether its incidence depends on *ERG* status. Similar fraction of cases in *ERG* negative and *ERG* positive subsets was shown to harbor chromothripsis suggesting that ERG is unlikely to contribute to the induction of either (Figure 2A).

The groups of PCa cases were also compared for the incidence of complex inter-chromosomal clusters. Unlike catastrophe, the frequency of complex inter-chromosomal clusters increased with a higher Gleason grade (Figure 1B). Forty one per cent of insignificant GS6, 64% of GP4 of GS7 and 72% of GS8+ cases demonstrated the presence of complex clustered breaks. When the GS7 group was split into GP3+4 and GP4+3 (Figure 2B), a difference between GP3 from GS7(3+4) and GS7 (4+3) was evident. The per cent of affected cases in GP3 from GS7(4+3) was closer to its counterpart GP4 and GP4 from GS7(3+4) than to GP3 from GS7 (3+4). These data suggest that inter-chromosomal clustered breaks begin to occur early, continue to accumulate as disease progresses and are likely to contribute to disease aggressiveness as they associate with the cancer grade.

### Correlative analysis of distribution of chromothriptic events and fragile sites

**Figure 3:**
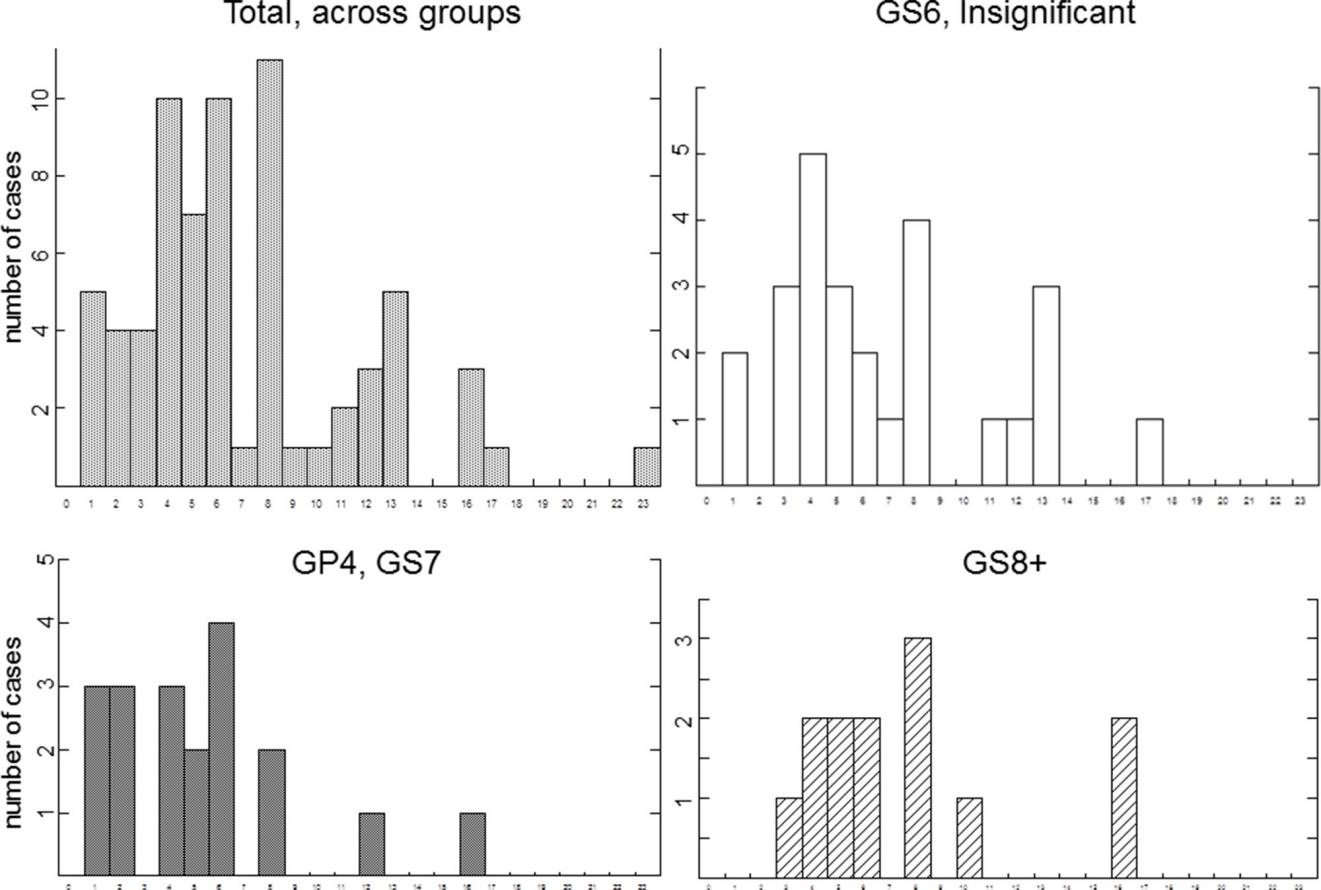
Comparison of distribution of catastrophic events among individual chromosomes Y axis shows number of affected cases, X axis shows affected chromosome. Groups of prostate cases are indicated.

We next examined whether distribution of breaks representing chromosomal catastrophe correlated with the distribution of fragile sites or correlated with the size of chromosomes. Common fragile site are known to be susceptible to breakage under conditions of replicative stress [29, 29] and are believed to contribute to carcinogenesis [26, 27]. We have compared the spread of the chromothriptic events in our set of samples to the distribution of common fragile sites using segment limits published earlier [30]. Our analysis did not reveal a strong correlation between distribution of fragile sites and that of chromothriptic events. For example, chromosome 16 was noted to harbor fragile sites every 9.89 kb [31] but did not appear to be involved in chromothripsis in many PCa, cases. Only three cases (one in GP4 of GS7 and two in GS8+) showed catastrophe at chromosome 16. Chromosome 2, shown to harbor the highest number of fragile sites, involving 21 regions [31], did not show higher incidence of chromothripsis as compared to other chromosomes comparable in size (Figure 3, left top panel).

Common fragile sites were reported to spread along each chromosome, and their number was shown not to depend on the size of chromosome [31]. We observed higher frequency of chromothripsis on larger chromosomes than on smaller (Figure 3). In fact, chromothripsis was never present on chromosomes 18–22, even in GS8+ group (Figure 3).

### Chromothripsis in adjacent Gleason patterns of the same tumor

**Figure 4:**
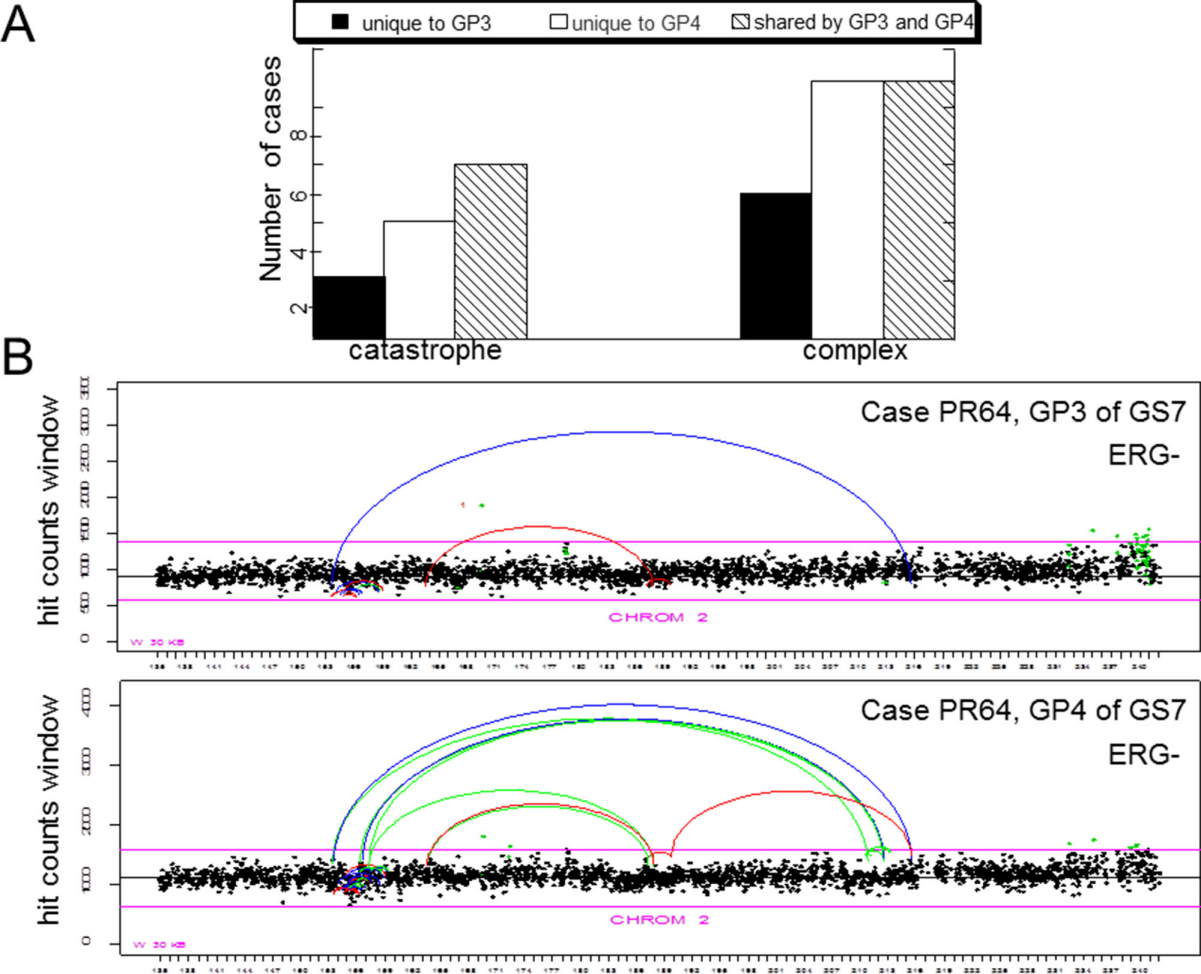
Comparison of incidence of chromothripsis and complex inter-chromosomal clustered breaks in adjacent GP3 and GP4 tumors **A.** Cases affected by chromothripsis and cases harboring inter-chromosomal clustered breaks (as indicated). Breaks unique to GP3, unique to GP4 of the same tumor or shared between the two are scored. **B.** Representative count plots illustrating presence of catastrophic event in both GP3 and GP4. Labels as in Figure 1A.

We have recently reported lineage relationship between cells comprising adjacent patterns 3 and 4 in GS7 PCa. In the present study, we scored and compared catastrophic events as well as clustered inter-chromosomal rearrangements between GP3 and GP4 regions of GS7 tumors (Figure 4A). Most of the cases where chromosomal catastrophe was present in the GP3, also had it in the associated GP4 (Figure 4B), consistent with lineage relationship between neighboring patterns of the same tumor reported earlier. A few cases showed chromothripsis only in GP3 that was not detected in GP4, thus suggesting that these tumors did not have lineage relationship [19]. Twice as many cases showed chromothripsis exclusively in GP4 but not in neighboring GP3 tumors suggesting that chromothripsis can occur at later stages. Validation of alterations within a chromothriptic event (Supplementary Figure S3) using PCR revealed the presence of the same chromothiptic breakpoints in the neighboring GP3 and adjacent normal but not distant normal cells (ref. 22 and Supplementary Figure S4), while bioinformatics analysis showed chromothripsis only in GP4 of this case (Supplementary Figure S3). The results are consistent with the notion that chromosomal catastrophe can occur at any stages of prostate tumorigenesis. Similarly, complex inter-chromosomal rearrangements can be present only in GP3, only in GP4 or can be shared between the two in the same tumor (Figure 4A), suggesting that they are likely to occur at any stage of tumorigenesis and can progressively accumulate over time.

## DISCUSSION

Chromothripsis is observed in both, DNA from tumor and germline DNA of patients with congenital diseases. The degree of chromothripsis varies in different cancers. Compared to other tumor types PCa demonstrates a high incidence of chromothripsis, affecting 30–45% of cases. Importantly, chromothripsis was observed in the same frequency in insignificant GP3 and GP3 associated with GP4. This is the first study to compare incidence of chromosomal catastrophe in a large set of PCa cases that includes clinically insignificant GS6 tumors and PINs. Unlike other cancers where chromothripsis was shown to associate with poor prognosis, PCa appears to be different. We find that chromothripsis in PCa does not affect genes involved in promotion of cell growth and aggressiveness. Our analyses reveal that genes, known drivers for prostate cancer, are altered independently from chromothripsis. The data rather suggest that alterations comprising chromothriptic cluster affect genes (Supplementary Table S3) that play role in cancer initiation.

Although, no correlation in the distribution was found between chromothripsis and fragile sites in this study, both can be dependent on replication and chromatin structure. The mechanism underlying fragility, chromothripsis and complex clustered rearrangements was proposed in multiple reports to be replication-dependent [1,32–34]. In addition, “hot spots” for chromothripsis may also be impacted by tissue-specific transcription. Recent study has reported that copy number variants resulting from deletions and duplications under replication inhibition frequently occur at the same loci in the given cell type and are enriched at transcribed large genes [35]. Whether chromothriptic events also affect such regions remains to be elucidated.

Fragile site-specific rearrangements are frequently observed in cancer. For example, proto-oncogene *c-myc* located at FRA8C is often amplified in cancer [36]. Likewise, *FHIT* and *WWOX* deletions within fragile sites FRA3B (most frequently expressed) and FRA16D, respectively, have been observed in many cancers [37, 38]. *c-Myc* gene is known to be amplified in PCa, contribute to prostate carcinogenesis and is associated with poor prognosis [39]. *Myc* amplification is specifically noted in castration-resistant tumors [24]. However, the 8q24.1 locus including *cMyc* was not affected by chromothripsis in our dataset, thus supporting the conclusion that there is no association between distribution of chromosomal catastrophe events and fragile sites.

It is not clear whether one homologous chromosome is affected by chromothripsis or both. Homozygous deletions were occasionally observed within chromothriptic alterations. Thus, it is possible that either the mechanism of repairing breakage after chromosomal shattering relies on the presence of homologous partner or chromothripsis itself involves two homologous chromosomes. Further studies are needed to resolve this issue.

There are distinct differences between the occurrence of catastrophic events and clustered inter-chromosomal rearrangements representing chromoplexy. The number of latter rises sharply in GS7 cancers, thus showing a correlation with more significant disease (Figure 1B). In tumors of GS7 and higher, these clustered rearrangements can be found in more than 70% of the cases suggesting that the alterations affect important genes that drive progression.

The fact that both chromothripsis and complex clustered rearrangements can be observed in less advanced cancer lesion GP3 within GS7 tumors but not seen in the same combination in the adjacent GP4 supports the hypothesis that these events can take place at different times and originate from different cell clones, some of which are selected and propagated, while others are not. The mechanisms underlying chromothripsis and complex inter-chromosomal clustered rearrangements may be shared or may differ. The former is more consistent with DNA shattering due to a replication fork collapse during S phase of the cell cycle following by non-recombinational repair. The latter, can also involve replication and may occur during chromosomal segregation at the mitotic phase and include recombination repair processes.

In conclusion, chromothripsis is a frequently observed phenomenon in PCa, it is present in clinically insignificant tumors and is not indicative of aggressive high risk disease.

## MATERIALS AND METHODS

### Prostate cancer samples

Frozen sections (10 um) of prostate cancer tissues (reviewed and graded for tumor content by a urologic pathologist) were dissected using Laser Capture Microdissection (LCM) system as previously described [19, 22]. Cells from adjacent normal glands, GP3 and GP4 tumors and high grade prostatic intraepithelial neoplasia (HGPIN) were collected separately.

### Next generation sequencing

Cell lysis and whole genome amplification was performed directly on LCM captured cells and library construction and mate pair whole-genome sequencing was carried out as described previously [22].

### Bioinformatics analysis of genomic breakpoints

A set of algorithms developed to detect large chromosomal aberrations (deletions, amplifications, inversions and translocations) was used [23, 40]. Briefly, the read-to-reference–genome–mapping algorithm consisted of 1) indexing the reference genome; 2) finding all possible mapping positions of both reads; and 3) aligning of the read pairs to find the optimal map position of the fragment. We used the protocol that allowed to sequence the ends of large fragments of genomic DNA (2.5–5kb), thus effectively covering breakpoints, by about 30× on average. Both bridged and base coverage were calculated. Breakpoints covered by at least 5 mate-pairs in each sample were collected for further analysis. Unmapped read-pairs (∼3–6% of all read-pairs) were removed from the data. Filters, based on homology scores calculated during mapping, were applied to eliminate false positives from the selection of events.

### Count plots

Plots of the counts of read-pairs mapped in non-overlapping consecutive 30 kb windows were generated to cover the entire genome. A masking operation using normal samples eliminated aberrant hit counts such as genomic regions not represented in the reference genome and regions with repetitive sequences. The mask was developed using independent normal samples treated the same as the cancer samples.

### Copy number variation (CNV)

CNVs in mate-pair data were identified by analyzing frequency distributions of window counts of mapped reads across the reference genome. The analysis was based on the assumption that tumors have a dominant (primary) mode in their frequency distribution corresponding to normal diploid areas of the genome and minor (secondary) modes or outliers that correspond to copy number alterations. The distribution of the primary mode was used to find windows with normal counts as well as windows with outlier counts. Thresholds for copy number gains and losses were determined by analyzing lower and upper secondary modes. Since outcome is dependent on the size of the window, iterative sizing moving from large to small window was used to detect large variations more accurately. Window sizes varied from 30-to-3,000 kb. Counts were calculated for the tumor sample and also for the normal sample, experimentally treated the same as the tumor sample. The correction vector from selected normal sample were then was used to normalize the count vector from the cancer sample to reduce the noise. In each iteration, a density distribution of the corrected counts was produced using the *density* function in R.

To call deletions and amplifications peaks and valleys of the frequency distribution were determined by finding the spots where the discrete derivative of the distribution would cross zero. The dominant mode was determined by finding the highest peak of that distribution which was also the maximum of the density function. The nearest left minimum was considered a threshold below which deletions were called. Amplifications were called by finding the neighboring minimum on the right of the highest peak.

### Filtering and masking methods for false positives

False positives calls in mate pair data are primarily due to artifacts of library assembly and germ line rearrangements. The two-step ligation mate-pair procedure [22] covers genome with untrue single and double chimeras that often cluster to 3 and 4 paired sequences that look like a rearrangement. Thus, the events with 3 or 4 associated reads were dismissed as untrue. Five or more associated reads were required to be called a cluster. Occasional untrue clusters within this group (can also occur) were removed by a devised filtering step that took into consideration the abnormal spreads and overly mixed orientations of the reads. Efficient algorithmic masks derived from the mate pair sequencing of 30 normal samples and over 1000 independent clinical tissues were used to eliminate the majority of the low level germline events. Since these germline rearrangements are often represented by smaller deletions/amplifications/inversions, they are flagged and filtered out by additional filter. Extensive validation experiments confirmed the false positive rate being less than 5, mostly 1–2 events in each sample.

### Comparison of distribution of fragile sites and chromothriptic events

The segment limits corresponding to HG18 were converted to HG19 coordinates using an R script that utilized the *liftOver* function and the hg18ToHg19.over.chain data. All breakpoints in prostate samples that were supported by at least six mate-pairs were stratified to three groups. The first group included all inter-chromosomal breakpoints. The second included all intra-chromosomal breakpoints that were not considered to belong to chromothripsis events. The third group included all intra-chromosomal breakpoints that were considered to result from chromothripsis events. The algorithm to predict breakpoints resulting from chromothripsis scanned the genome of each tumor sample for areas that were shuffled 5 times or more in each sample. All breakpoints involved in shuffling (at least 10, corresponding to 5 rearrangements) were flagged for chromothripsis. The three groups of breakpoints were then interrogated for hitting within or outside common fragile sites. In all three groups the fraction of breakpoints that hit within boundaries versus those that hit outside was not different than that of by chance alone.

## SUPPLEMENTARY FIGURES AND TABLES


