# Supplementary material for: Chromosomal catastrophe is a frequent event in clinically insignificant prostate cancer
Source: Oncotarget. 2015 Aug 21;6(30):29087–96. doi: 10.18632/oncotarget.4900 (PMC4745713; doi:10.18632/oncotarget.4900)
Supplement: Supplementary file 1 [file oncotarget-06-29087-s001.pdf]

SUPPLEMENTARY INFORMATION

Validation of selected rearrangements within chromothriptic event

Mate pair sequence reads for five events within chromothripsis on chromosome 4 (Supplementary Figure S3) for case PR6 were mapped to the human genome, and primers spanning the fusion junctions (sequences published in Murphy et al 2012, ref. 22) were used in validation PCRs as was previously described. DNA for PCRs was isolated from cells collected separately from GP3 and GP4 tumors, adjacent and distant normal tissue using laser capture microdissection. Amplification products we resolved on the gel (Figure 4 in Murphy et al, 2012, ref. 22), extracted and Sanger sequenced (below). The structure of rearrangements based on the sequence of amplified products is shown in Supplementary Figure S4.

Sanger sequencing results of validated rearrangements

The sequences of two joined pieces (rearranged) and their corresponding chromosomal positions are listed below.

Rearrangement I

4 + 121954855 121955371
4 + 126358698 126358904

1. CCCNNTNNNCNTTAGCTGTTTTGNNNNG
ANANCTTTTACNAATTTATTCTGACTTCTATGATCT
TTAATCTCTTTTCTACACCTGAAAAATAATAAAGA
TTGTAAACTAGATGTCCTCAAAGATTCTTCCCTCC
CCAGTGATACATGACTATACTTGGGACTCTCTAAA
TGCCCTGCTGTCAACAGAGCTATCCTGAAGCTTA
CTGAACACAAGGTGGTGCTTTCATATGAGCTTCT
TTCTAGTTCTTTTGTTAATTAAGAACAAGTTTTTG
TTTTTTGTCCCCAGTTTAGCCAAATGCTCCTGAAA
TAGAAATGCATTAGTAAATAAGAGCTTCATTTTTA
ATAGGTTTTCCCCTTTCTTTAGGAGTGATAGAAGT
TTCATAATGTGTATTTCGTTTTCCTAATAATTTCAA
AAAGGCAAATTTCACTAGGGCCATGAAAGTATTC
TTGGATTTATCCAGACTATGTA
GAAACCACCATCACAGCTGGCTAAAAAGCAAAA
GCTTGGTTAAATTGACTTGGCGTGG
2. ACTGAGAAATTTCTGTCCAGGGTCATAAC
CCGTTTGAGTCATTGTACTTAGCACCAACCAGAG
AGAGGCAGGTACAGCTGANCTGCTTCCAGGCTAT
GAGCTGCACAANNNCAGTCAATNAANCCCCTTTT
ACTTGTCTTATNCCANAGCAAAGANATANAAANA
ATAAAANTCAAANAATAATATTCAATCCAAAGTC
TNAGCTTANCAGTNGGAGTANACCNTGNNNATN
AANTTGTGTGAANTNAATCCTGANTNTACATTTN
CTNTNGCCTTANNNTGNGCGNNNNNNNANTNTC
TGGACCNNGTTTNAGGGNATTAGC

Rearrangement II

4 – 102288117 102288776
4 + 145213837 145214159

1. ANNNTGNGNATCTCCCTTCTCTCCNTGGA
ATGGAGAGTGAACTGGAGTTTGGTTTTCCCTTCCC
CTAAGTCAGTTGGCTTGATAAAACACTAGCAGATT
AGGCTCTGGTTAACCAGTTTCTCTTAAGAGCATAC
TTTGTTAAGAAAAACAGACTAGTGAGAATCTGGT
TGAGTTCCTGGAGATAAACCTCACAAAAATGTGG
AGGCCTCCCTGTGACTGGGTTTGTCTGGAATTTTT
AACTTTCATACTTGAGCAATTAGTAGGTTACA GTT
CAGGTTTTTCTACCCTAGCACTGCTTCCCGTAGAG
TTTTCTGCTGTGATATGTTGTCATTTTTCTGTATCT
GTCTGTCTGTCTCTCAAGTTTGGGGGCATCAGTTT
TTCCTTTGACCTCAATTCTCTTAAAAATCCAGCAA
CTGTTGATTTTTTCAGTTTGTTCAACCTTTTACTTG
CTGTCAGGACAGAGTAAAGATTTCTTTCTTTCTT
TTTTTTCTTTTTTTTCTTTTTTTTTGACGGAGTCTT
GCACTGTCACCCAAGCTGGAGTGCCGTGGCGTGA
TCTCAGCTCACTGCAAGCTCCGCCTCCAGGGTTC
ACACCATTCTCCTGCCTCAGCCTCCCAAGTAGCT
GGGACTACAGGCGCCCGCCACCACGCCTGGCTAA
TTTTGTGTGTGTG
2. GAGAATATTTCTTTGTAATGAATAAATGAA
CGTTAAAAATAAGCTAAGTATACCAAGAAGCAAA
TAAATCAAATTAAATTTGGCTGAAAAGAAGTTTAT
AAAATTGACATTCCCCCTAAACTAGCCTTTAAATT
ACTAAAAAAAGACATAAATGCAAACATCACGTANA
GTGAATAACCNACACTGAGTGTCAGTTCTTTCTAG
CTGTTTAGGTATGTGNNAATGAAATGACCCATGGA
TAANAAAAATACACACNTTGGTTCTGTAATGTGCA
CTAAATAATCTGAANCNNGGGCNGCCACACAAGT
TCTAACACTTTGTGTTGNACACNNGGGNTTCCNC
GGAAAAGGGGTNTAAGAANGGCACCA

Rearrangement III

4 + 146812565 146813253 689
4 – 121967662 121967956 295

1. TGTCTTTGACAACACCCCTACAGCAATGT
GCAGTAGAGAATGATCCATCATTACTTAGAGGATC
TTTGATGAAAGTCAGTGACAAATGCCAAAATGTA
ACTCAGTGAAAGTACTCTCTGCTGGAGGTTAAGA
CAAATGTTCTAAATATACACCTTTCTGGCTTGATGT
GTGAGTGAAGGTTCAAGCCCATTTTCTAAAATGA
TAGAGGTGTATATGTCAACGTTGTGTTGAAGACTC
TGGTACCTGATAACAACTAAACATCAACTAATAAC
TCATCTTGTGATTCTGGACAAAT

2. AGTTTCCTCCTGGATTTTGAGACAAAACC CTTTGCATTAACGGAGTCAGCTAAAAAGATTTCC ATCTAACTAAGTTTTCCCCCATCTCATATAAGAGG GACTCATGGATTCCAGTCAATTTTTCTGTACATGT TTAGTGTAGGTTTGAGCTCCCCAGTTGTTTTTAAT AGGACATTGAAAATTATGTCCTTGGATGTTTGGTA AATGTAATCCTGGGAATTATGATAGCTCAAACAAT GACAGCCTCAATGAGGACTACAGTCTTGTTAGAA AGGGCCCCCAAGCCTCCCACAGGGCTAAATAAA GTTAAAGGCCCGGTGCCTTCACTGTCTTCATGCC TCCTACACGGTGTCTCTGTGTGTTCCTCTTAGTTT GCAAGCTGGAGGGTAAGGCAGTAACAGCCAAGG GCAGTCTCTCCAGGAAGGGGAGCAGGTTCAACA GCCCTCTCTTTGGTGCTCAGTCCAGCAGCTCTTC CTGAACTTGGCTTTCCCTGCTTCTTGCTATTCCCC AGCCCNTGCTTCTGAGTGCCCCAAGAAATGCGTC ANCNCACAGGCCAGGCAGGCTGTTGCTCCNCTAT AGGAGAGGACCGCCCTNCAGTAAATACATNATTT GGATATCGAAGGTCTNTATGTCTGCCTTAAGNTCT TGGCCTGAAAGTGGACATTACAGCTTTCTGNTGA ATTCTACTTTTGTACCTTGTAGTACTGNNANCCNN ANGTTTCATCAATNGAGCNCNCANCNNTTCNCAA

Rearrangement IV

4 – 101807349 101808165 817
4 – 137883309 137883499 191

1. AACNNGNATAANNAATANCNGCAANCCN ANGACGTTNAGCACNGTACACAATAGATAAGATN TGGAAGCAATCTAAGTGTCCATCAGCAGATGAAT GGANAAAAAAATGTGATGCTTATACAAAATGGAG TACTATTGAGCCAAAATAGTTAAAACTGCAGTTA CATCAGCATGAATCAAAGTGGAGACACCAACTGT ACGTGTAGTCAATGCATTCTTCACTGC

2. AACACTGGAGCACAGATATATAAAGCAAA TATTATTAGAGCTAAAGAGAGAGATAGGGCCCAA TACAATACTAGCTGTAGACTACAACACCCTACTTT CAGCATTGGACAGATCTCCCAGACAGAAAATCAA CAAAGAAACAACAGACTTAATCTGCAATATAGAC CAAATGGACCTAACAGATACTTATTGAACATTTGA ACATTTCATCCAATGGCTGCAGGTTACAAATTCTT TTCCTCAGCACATGGATCATTGTCAAGGATAAATC ATGTGTTAAGTCACAAAACAAGTCTTCAAACATT TAAAAATAGTGAAATAATATGAAGCATCTTCTCTG ACCACAATGGAATAAAACTAGAAATCAATAACAA GAGGAATTTTGGAAACTATACCAACACATGGAAG TTAAACCATATGCTCCTGAATAACCAGTAGGTCAA TGAAAAAATAAAGAAAGAATTGAATAAATTTATT G AAACAAATGATAATGGAAACACAGCATATCAAA A CCTGTGGAATACAGCAAAAAAAGGACTAAGAGG GAAATTTACAGCTATAAGTACCTACATCAAAAA A GAAGAAAAACTTCAAATGAACAACCTAACAAT GC ATCTTAAATAGCTNGAAAAGCAAGAGCAAACA AA CTCAAATATAGTAGAAGAAAATAAATAATAAAT AT CAGAGCAGAGATAACTTAATTTGACNTGANTA AA ACAATACAAAAGATCAATAAAATGAAGTTTGT TT TTCANAAAGATAAACAAAANTTGANCAANCT GTN GATGGACTAANNAAAAAAAGAGGGAAGACTCA

Rearrangement V

4 + 107030113 107030327
4 + 146127590 146127768

1. TTGTATTNGTCTGTTGTCTGCTGCTAATAA AGATTTACCTGAGACTGGGTAATTTATAAAGGAA AGAAATTTAATGGGCTCACACATTCACATGGCTG GGGAGGCCTCACAATCATGGCAGAGGCAAAGGG GAAGCAGAGGTACATCTTACATGGTGGCAGGCAA GAGAGCT

2. CAACGACAACCAACCTGAAAGCAAAATC AGAAAGGCAATCCCATTCACAACTGACACACACA TACACACACACACACACACACACACACACACTCC CTAGGAATACAGCACACCGGGGGGGTGAAGGAC CCCTACAATGAGAATTACAAAACACTACTCAAAG AAATCAGAAAAGACACAAACAAATGGAAAGACA TCCCATGCTATGGATCGGAGGGNNCAANANAAG

SUPPLEMENTARY FIGURES AND TABLES

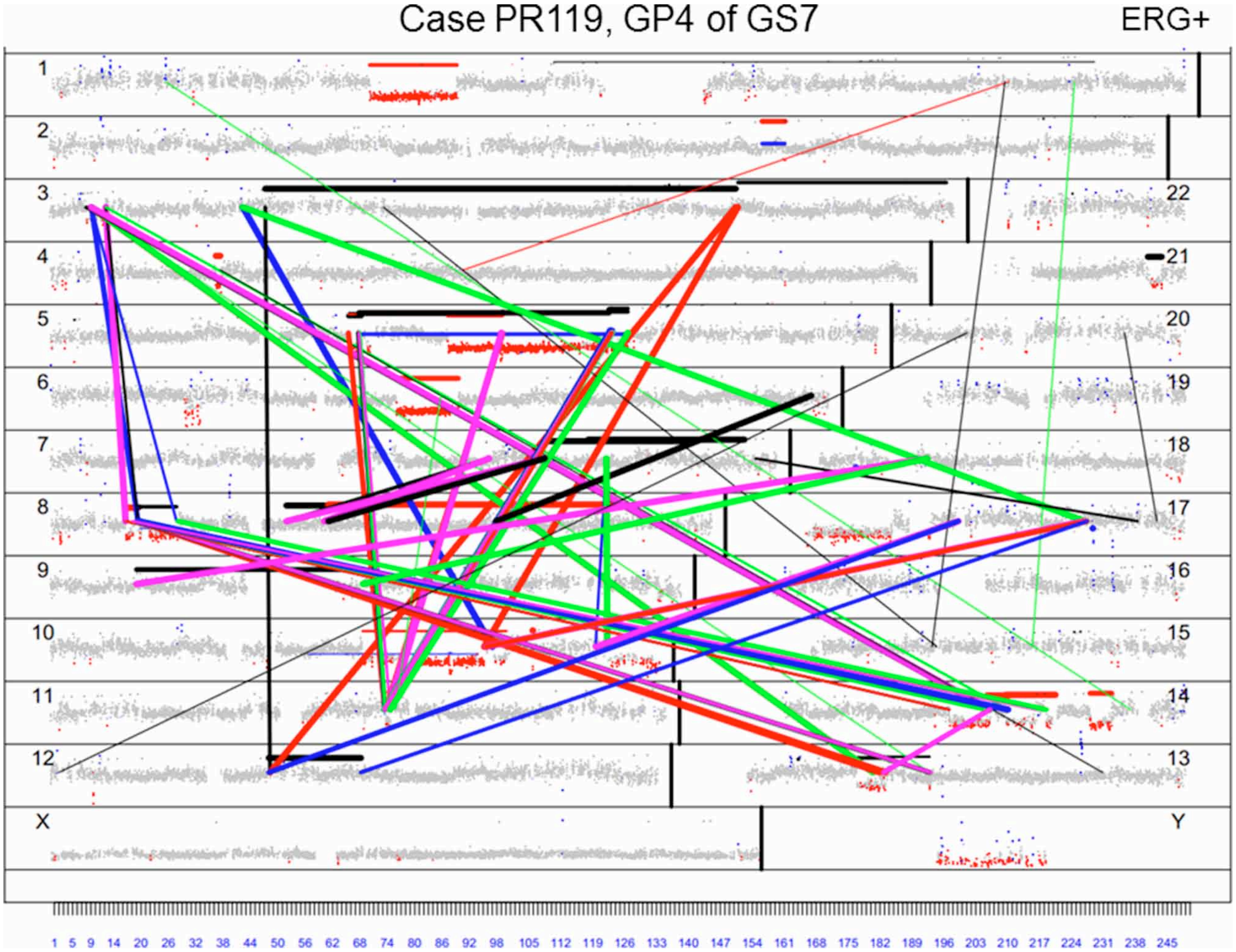

Supplementary Figure S1: Genome plot of rearrangement landscape in representative prostate cancer case. Count plots show frequency of distribution of reads in 30KB windows and breakpoints for all chromosomes (numbers are indicated). The X axis spans the length of the chromosome, the Y axis shows the number of reads for each window. Window counts are shown points colored according to the prediction of CNV algorithm. Black points are normal, red points correspond to deletions and green points show gains. Lines connect identified bioinformatically breakpoints. The widths of the lines correlate with number of associated mate-pair reads. Color of the connecting lines indicate polarity of the joined chromosome. For intra-chromosomal events red shows forward direction for both pieces, green indicates inversion for one partner and blue shows inversion for both. For inter-chromosomal events, red connects the p-side piece from the larger chromosome to the q-side piece of the smaller chromosome in forward direction, green connects the q-side piece from the larger chromosome to the p-side piece of the smaller chromosome in forward direction, blue connects the p-side piece from the larger chromosome to the p-side piece of the smaller chromosome in reverse direction and magenta connects the q-side piece from the larger chromosome to the q-side piece of the smaller chromosome in reverse direction. Black indicates balanced translocations.

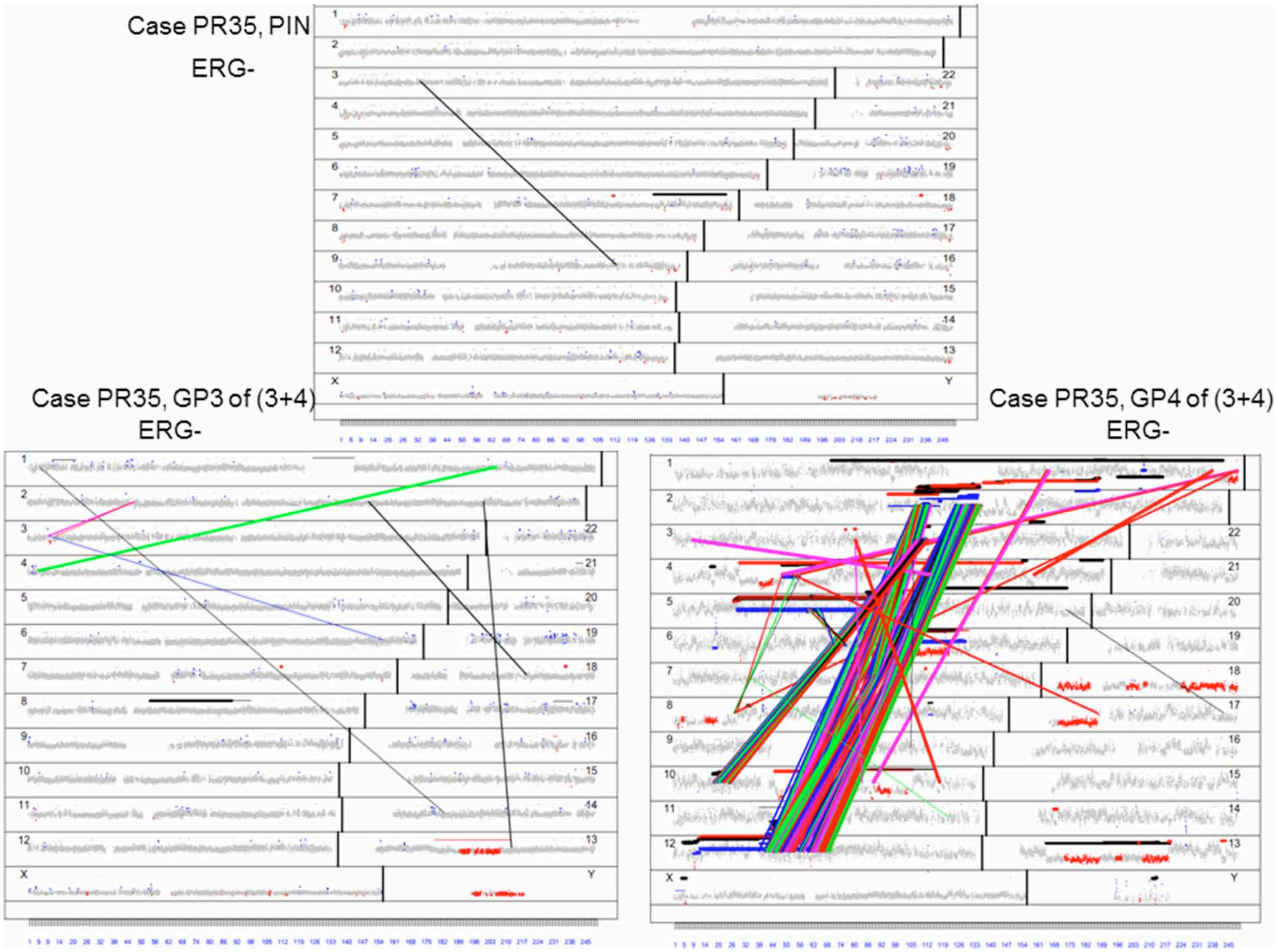

Supplementary Figure S2: Genome plots of rearrangement landscape in representative prostate cancer case. Count plots for PIN, GP3 and GP4 tumors of the same prostate cancer case. The designations are the same as in Supplementary Figure S1.

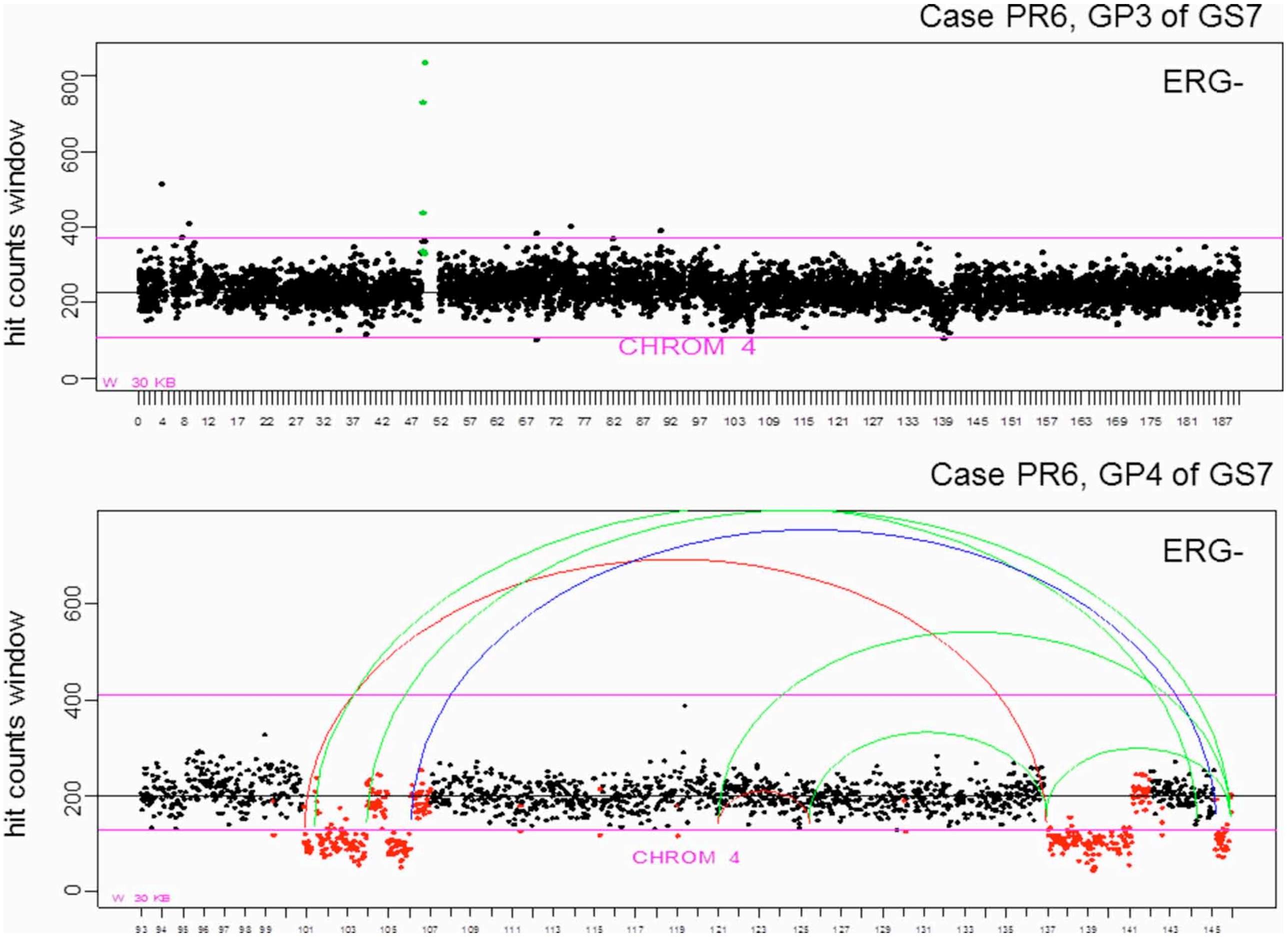

Supplementary Figure S3: Representative count plots for adjacent GP3 and GP4 tumors. Chromothripsis is present in GP4 (bottom) of GS7 (3+4) case (zoomed in area of chromosome 4) and absent in adjacent GP3 tumor (spans entire chromosome, top). Count plots show frequency distribution of reads in 30KB windows and breakpoints for indicated chromosome. The X axis spans the length of the chromosome, the Y axis shows the number of reads for each window. Window counts are shown by points colored according to the prediction of CNV algorithm. Black points are normal, red points correspond to deletions and green points show gains. Color of the connecting loops indicate polarity of the joined chromosomal pieces: red shows forward direction (concordant) for both pieces (represents deletions), green indicates switch in polarity (represents inversion) and blue indicates change in direction (gain). ERG status is indicated.

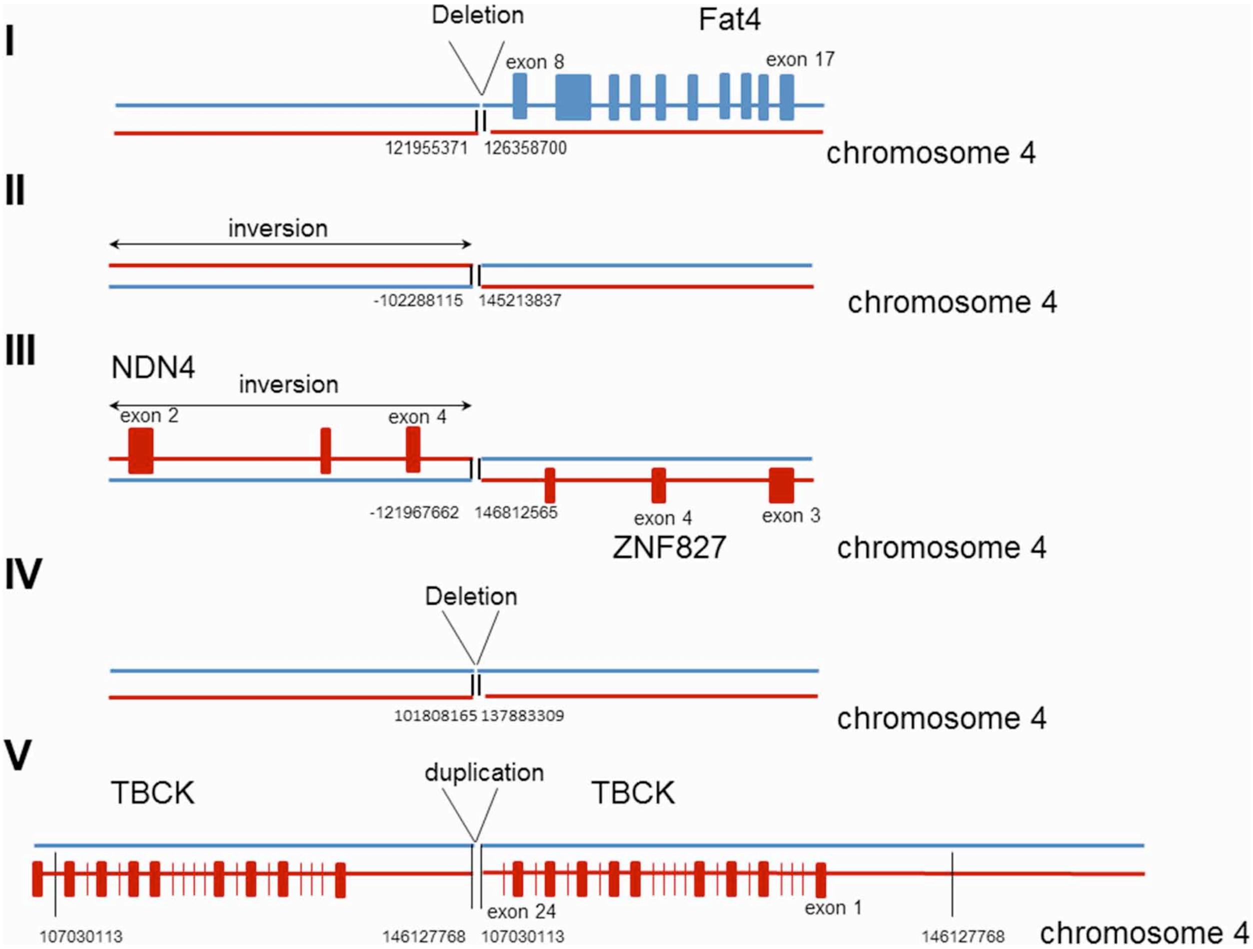

Supplementary Figure S4: Schematic of validated rearrangements within chromothripsis for the case shown in Supplementary Figure S3. Validation analysis of five selected breakpoints (designated I–V) identified by mate pair in GP4 of PR6 case. Representative gel images PCR products through selected breakpoints were published previously [22]. The schematic of validated breakpoints is based on Sanger sequencing results (described in Supplementary materials). Blue color codes for "+" strand, red color codes for "–" strand in the reference genome, exons are shown as boxes. The chromosomal positions are indicated by numbers. The genes that a "hit" are listed. Additional genes that have been deleted (in rearrangements I and IV) are presented in the Table S3.

Supplementary Table S1: Chromothripsis and clinical outcome in prostate cancer

Group	Years follow up Mean+/− SD (n)	# cases Systemic Progression (n, chromothripsis)	# cases Systemic Progression (complex)	# cases PCa death (chromothripsis)	# cases PCa death (complex)
**GS6 insignificant**	8.1 + 2.38 (31)	0	0	0	0
**GS6 Large volume**	6.54 + 1.3 (22)	0	0	0	0
**GS7 (total)**	7.86.31 + 3.96 (16)	4(1)	4(3)	0	0
**GS7 (3+4)**	5.88 + 5.81 (9)	3(1)	3(2)	0	0
**GS7 (4+3)**	7.1 + 2.35 (7)	1(0)	1(1)	0	0
**GS8+ (total)**	7.5 + 1.2 (23)	7(4)	7(5)	3(1)	3 (3)

Supplementary Table S2: Quantification of chromothriptic events across all Gleason grades

Group	Number of caseswith 2 hits*	Number of caseswith 3 hits*	Number of caseswith 4 hits*
Insignificant, GS6	4	1	0
Large volume, GS6	1	1	0
GP3 of GS7	2	1	0
GP4 of GS7	3	1	1
GS8 and GS9	4	0	0

*Hit represents number of chromosomes on which catastrophe is observed

Supplementary Table S3: List of affected by chromothripsis (Supplementary Figure S3) genes in case PR6

Gene name	Gene name	Gene name
ABCE1	HSPA4	SGMS2
ADAD1	IL15	SLC10A7
AGXT2L1	IL21	SLC7A11
AIMP1	INFP4B	SLC9B1
AK2	LARP1B	SLC9B2
AK2	LARP7	SMARCA5
ALPK1	LEF1	SMS2
ANAPC10	LFCDH10	SPATA5
ANKRD50	LRIT3	SPRY1
ANXA5	MAD2L1	SYNPO2
AP1AR	MAML3	TACR3
ATHGEF38	MANBA	TBCK
BANK1	METL14	TBC1D9
ARSJ	MFSD8	TET2
BBS12	MGARP	TIFA
BBS7	MGST2	TMEM155
BDH2	NAA15	TNIP3
CAMK2D	NDNF	TRAM1L1
CASP6	NDST4	TRPC3
CCRN4L	NDUFC1	UBE2D3
CENPE	NEUROG2	UCP1
CFI	NFKB	UGT8
Col25A1	NPNT	USP38
CXXC4	NUDT6	USP53
CYP2U1	OSTC	
DKK2	OTUD4	

(*Continued*)

Gene name	Gene name	Gene name
EGF	PAPSS2	
ELF2	PCDH18	
ELMOD2	PDE5A	
ELOVL5	PGRMC	
ENPEP	PHF17	
EXOSC9	PITX2	
FABP2	PLA2G12A	
FABPC4L	PRDM5	
FAT4	PRSS12	
FGF2	QRFPR	
FLK4	RAB33B	
FREM3	RNF150	
GAB1	RPL34	
GAR1	RRH	
GSTCD	SCL1T	
GYPA	SCOC	
GYPB	SEC24B	
GYPE	SEC24D	
HADH	SETD7	
HADH	SETD7	
